# Oral β-Lactam Antibiotics vs Fluoroquinolones or Trimethoprim-Sulfamethoxazole for Definitive Treatment of Enterobacterales Bacteremia From a Urine Source

**DOI:** 10.1001/jamanetworkopen.2020.20166

**Published:** 2020-10-08

**Authors:** Jesse D. Sutton, Vanessa W. Stevens, Nai-Chung N. Chang, Karim Khader, Tristan T. Timbrook, Emily S. Spivak

**Affiliations:** 1 Department of Pharmacy, Veterans Affairs Salt Lake City Health Care System, Salt Lake City, Utah; 2 IDEAS Center of Innovation, Veterans Affairs Salt Lake City Health Care System, Salt Lake City, Utah; 3 Division of Epidemiology, Department of Internal Medicine, University of Utah School of Medicine, Salt Lake City; 4 now at BioFire Diagnostics, Salt Lake City, Utah; 5 Division of Infectious Diseases, Department of Internal Medicine, University of Utah School of Medicine, Salt Lake City

## Abstract

**Importance:**

Oral β-lactam antibiotics are traditionally not recommended to treat Enterobacterales bacteremia because of concerns over subtherapeutic serum concentrations, but there is a lack of outcomes data, specifically after initial treatment with parenteral antibiotics. Given the limited data and increasing limitations of fluoroquinolones or trimethoprim-sulfamethoxazole (TMP-SMX), oral β-lactam antibiotics may be a valuable additional treatment option.

**Objective:**

To compare definitive therapy with oral β-lactam antibiotics vs fluoroquinolones or TMP-SMX for Enterobacterales bacteremia from a suspected urine source.

**Design, Setting, and Participants:**

A retrospective cohort study was conducted from January 1, 2007, to September 30, 2015, at 114 Veterans Affairs hospitals among 4089 adults with *Escherichia coli*, *Klebsiella* spp, or *Proteus* spp bacteremia and matching urine culture results. Additional inclusion criteria were receipt of active parenteral antibiotic(s) followed by conversion to an oral antibiotic. Exclusion criteria were previous Enterobacterales bacteremia, urologic abscess, or chronic prostatitis. Data were analyzed from April 15, 2019, to July 26, 2020.

**Exposures:**

Conversion of therapy to an oral β-lactam antibiotic vs fluoroquinolones or TMP-SMX after 1 to 5 days of parenteral antibiotics.

**Main Outcomes and Measures:**

The main outcome was a composite of either 30-day all-cause mortality or 30-day recurrent bacteremia. Propensity-based overlap weights were used to adjust for differences between groups. Log binomial regression models were used to estimate adjusted relative risks (aRRs) and adjusted risk differences (aRDs).

**Results:**

Of the 4089 eligible patients (3731 men [91.2%]; median age, 71 years [interquartile range, 63-81 years]), 955 received an oral β-lactam antibiotic, and 3134 received fluoroquinolones or TMP-SMX. The primary outcome occurred for 42 patients (4.4%) who received β-lactam antibiotics and 94 patients (3.0%) who received fluoroquinolones or TMP-SMX (aRD, 0.99% [95% CI, −0.42% to 2.40%]; aRR, 1.31 [95% CI, 0.87-1.95]). Mortality rates were 3.0% (n = 29) for patients receiving β-lactam antibiotics vs 2.6% (n = 82) for those receiving fluoroquinolones or TMP-SMX (aRD, 0.06% [95% CI, −1.13% to 1.26%]; aRR, 1.02 [95% CI, 0.67-1.56]). Recurrent bacteremia rates were 1.5% (n = 14) among those receiving β-lactam antibiotics vs 0.4% (n = 12) among those receiving fluoroquinolones or TMP-SMX (aRD, 1.03% [95% CI, 0.24%-1.82%]; aRR, 3.43 [95% CI, 0.42-27.90]).

**Conclusions and Relevance:**

In this cohort study of adults with *E coli*, *Klebsiella* spp, or *Proteus* spp bacteremia from a suspected urine source, the relative risk of recurrent bacteremia was not significantly higher with β-lactam antibiotics compared with fluoroquinolones or TMP-SMX, and the absolute risk and risk difference were small (ie, <3%). No significant difference in mortality was observed. Oral β-lactam antibiotics may be a reasonable step-down treatment option, primarily when alternative options are limited by resistance or adverse effects. Further study is needed because statistical power was limited owing to a low number of recurrent bacteremia events.

## Key Points

**Question:**

Are mortality and recurrent bacteremia different for patients who receive oral β-lactam antibiotics vs fluoroquinolones or trimethoprim-sulfamethoxazole for definitive treatment of Enterobacterales bacteremia from a urinary source?

**Findings:**

In this cohort study of 4089 patients, the relative risk of recurrent bacteremia at 30 days was not significantly higher with oral β-lactam antibiotics than with fluoroquinolones or trimethoprim-sulfamethoxazole, and the absolute risk difference was small.

**Meaning:**

This study suggests that oral β-lactam antibiotics may be a reasonable definitive treatment when alternatives are limited by resistance or adverse effects.

## Teaser

This cohort study compares definitive therapy with oral β-lactam antibiotics vs fluoroquinolones or trimethoprim-sulfamethoxazole after 1 to 5 days of parenteral antibiotics for Enterobacterales bacteremia from a suspected urine source.

## Introduction

Enterobacterales bacteremia occurs in up to 20% of patients hospitalized with pyelonephritis or systemic urinary tract infections (UTIs).^1^ Guidelines do not directly address the use of oral antibiotics in the setting of Enterobacterales bacteremia, but several experimental and observational studies support a “step-down” from initial parenteral therapy to definitive oral therapy, especially with bacteremia from a urinary source.^2,3,4,5,6^ Patients in these previous oral step-down studies primarily received fluoroquinolones or trimethoprim-sulfamethoxazole (TMP-SMX),^4,5,6^ which result in similar serum concentrations whether given via parenteral or oral routes. Therefore, fluoroquinolones, TMP-SMX, and parenteral antibiotics are considered the mainstays of definitive treatment.^6,7,8,9,10,11^ These mainstays are increasingly limited by antibiotic resistance rates, adverse effects, cost, or decreased patient satisfaction.^12,13,14^ Additional oral antibiotics, if effective, would be a valuable addition to step-down treatment options.

Oral β-lactam antibiotics are not routinely recommended as step-down therapy for Enterobacterales bacteremia owing to concern over subtherapeutic serum concentrations.^8,15^ Although potential subtherapeutic concentrations may legitimately preclude their routine or empirical use before organism identification and susceptibility determination, their role as step-down therapy on an individual patient basis is understudied, to our knowledge.^11,16^ Given the limited available data, our objective was to compare rates of mortality and recurrent bacteremia in patients treated with β-lactam antibiotics vs fluoroquinolones or TMP-SMX as oral step-down therapy for Enterobacterales bacteremia from a suspected urinary source.

## Methods

We conducted a retrospective cohort study of patients treated for Enterobacterales bacteremia at Veterans Affairs (VA) hospitals from January 1, 2007, through September 30, 2015. The exposure was oral step-down treatment with a β-lactam antibiotic compared with fluoroquinolones or TMP-SMX. Patients were included if they met all of the following criteria: (1) had matching blood and urine cultures with *Escherichia coli, Klebsiella* spp, or *Proteus* spp; (2) were hospitalized; (3) were aged 18 years or older; (4) received in vitro active parenteral empirical antibiotics for 1 to 5 days; and (5) had their therapy transitioned to a single oral β-lactam antibiotic, fluoroquinolone, or TMP-SMX by the sixth day of antibiotic treatment. Exclusion criteria were polymicrobial bacteremia, diagnosis of a urologic abscess or chronic prostatitis in the 90 days before blood culture collection, or *E coli*, *Klebsiella* spp, or *Proteus* spp bacteremia in the 365 days before blood culture collection. The study was reviewed and approved by the University of Utah Institutional Review Board and the Research and Development Committee of the VA Salt Lake City Health Care System, who waived patient consent because the project relied on retrospective analysis of existing patient records. The study followed the Strengthening the Reporting of Observational Studies in Epidemiology (STROBE) reporting guideline and checklist for cross-sectional studies.

*Klebsiella aerogenes*, which was *Enterobacter aerogenes* during the study period, was not included among *Klebsiella* spp. Eligible urine cultures were those collected within 24 hours of blood culture collection. Hospitalization was defined as presence in an acute care unit within 1 calendar day of blood culture collection. Active parenteral empirical antibiotics were those received within 48 hours of blood culture collection and reported as susceptible in the medical record. Any of the following were eligible empirical antibiotics: aminoglycosides, β-lactam antibiotics, β-lactam and β-lactamase inhibitors, fluoroquinolones, polymyxins, or TMP-SMX. Antibiotic day 1 was defined as the first calendar day an in vitro active parenteral antibiotic was received.^6,17^ The day of oral antibiotic step-down was the first calendar day the patient received an oral antibiotic alone, without documented receipt of a parenteral antibiotic with gram-negative activity. This definition corresponds to patients receiving a parenteral antibiotic on up to 5 calendar days before oral antibiotic step-down.

### Outcomes

The primary outcome was a composite of either 30-day all-cause mortality or 30-day recurrent bacteremia starting from day 1 of oral step-down therapy. Recurrent bacteremia was defined as a blood culture result with the same genus and species, if reported, as the initial positive blood culture result. Secondary outcomes were 90-day mortality, 90-day recurrent bacteremia, and repeated hospitalization with a UTI within 30 and 90 days. Repeated hospitalization with a UTI was defined as a new hospitalization after the original discharge date in which there was a UTI diagnosis code, receipt of gram-negative antibiotics, and a urine culture growing the same organism genus as the initial positive blood culture result (eTable and eAppendix in the Supplement). Switching back to a parenteral antibiotic, for any reason, after day 1 of switching to an oral antibiotic and during the initial antibiotic treatment course and time to primary outcome were also reported as descriptive measures.

### Study Data and Data Analysis

All data were collected retrospectively from the VA’s Corporate Data Warehouse. Inpatient antibiotic data were obtained from the barcode medication administration system. Outpatient antibiotics were identified using pharmacy prescription fill data in the time frame after positive blood culture results. Outpatient prescriptions were assumed to be taken as prescribed and to begin on the pharmacy fill date or the hospital discharge date, whichever was later. If no VA outpatient antibiotic prescription fill was recorded, discharge summaries and progress notes were manually reviewed for non-VA outpatient antibiotics.

Baseline comorbidities were extracted using the Charlson Comorbidity Index–Elixhauser score in the 365 days before blood culture collection.^18^ Urologic comorbidities were extracted based on *International Classification of Diseases, Ninth Revision, Clinical Modification* diagnosis codes in the 90 or 365 days before blood culture collection, as specified (Table 1; eTable in the Supplement).^19^ Urologic procedures were defined by *International Classification of Diseases, Ninth Revision, Clinical Modification* procedure codes and *Current Procedural Terminology* codes in the 90 days before oral step-down therapy (eTable in the Supplement). Immunosuppression was defined as any history of solid organ or hematopoietic stem cell transplant, any daily maximum serum leukocyte count less than or equal to 1000 cells/μL (to convert to ×10^9^/L, multiply by 0.001) between blood culture collection and start of oral step-down therapy, use of immunomodulatory medication in the 90 days before blood culture collection, or use of high-dose corticosteroids (ie, ≥20-mg prednisone equivalents) for at least 14 of the 30 days before blood culture collection. Organism identification methods, susceptibility testing, breakpoints used, and reporting were heterogeneous and at the discretion of individual hospitals during the study period. Race/ethnicity, as self-reported by patient or their proxy during VA enrollment, was reported for descriptive purposes. Data were extracted from January 17, 2017, through November 29, 2019, and analyzed from April 15, 2019, through July 26, 2020. Data were accessed through the VA Informatics and Computing Infrastructure.

**Table 1.** Demographic, Clinical, and Treatment Characteristics

Characteristic	Patients, No. (%)	
	Fluoroquinolone or trimethoprim-sulfamethoxazole (n = 3134)	β-Lactam antibiotics (n = 955)
Age, median (IQR), y	69 (62-80)	73 (64-83)
Male	2847 (90.8)	884 (92.6)
Race/ethnicity		
White	1983 (63.3)	617 (64.6)
Black	714 (22.8)	202 (21.2)
Hispanic or Latino	185 (5.9)	53 (5.5)
Native American, Alaskan, Hawaiian, or Pacific Islander	41 (1.3)	13 (1.4)
Asian	20 (0.6)	3 (0.3)
Missing, unknown, or declined to answer	191 (6.1)	67 (7.0)
Preexisting conditions^a^		
Combined comorbidity score, median (IQR)	1 (0-2)	1 (0-3)
Chronic kidney disease	564 (18.0)	227 (23.8)
Chronic pulmonary disease	681 (21.7)	220 (23.0)
Heart failure	480 (15.3)	170 (17.8)
Diabetes with complication	402 (12.8)	130 (13.6)
Dementia	180 (5.7)	69 (7.2)
Immunosuppression	182 (5.8)	61 (6.4)
History of organ or stem cell transplant	72 (2.3)	22 (2.3)
Transplant antirejection medications within 90 d	64 (2.0)	20 (2.1)
High-dose corticosteroids within 30 d	39 (1.2)	17 (1.8)
Other immunosuppressive medication within 90 d	68 (2.2)	23 (2.4)
Leukopenia, leukocyte ≤1000 cells/μL	5 (0.2)	1 (0.1)
Metastatic cancer	144 (4.6)	47 (4.9)
Cirrhosis	88 (2.8)	20 (2.1)
HIV	40 (1.3)	10 (1.0)
Preexisting urologic conditions^a^		
History of urinary tract infection	886 (28.3)	401 (42.0)
Previous antibiotics active against gram-negative organisms within 30 d	398 (12.7)	222 (23.2)
Prostate hypertrophy	887 (28.3)	324 (33.9)
Urinary retention, obstruction, or other structural urologic abnormality	723 (23.1)	288 (30.2)
Urologic procedure within 90 d before oral step-down therapy	562 (17.9)	212 (22.2)
Prostate cancer	408 (13.0)	143 (15.0)
Spinal cord injury, paraplegia, quadriplegia, or multiple sclerosis	129 (4.1)	52 (5.4)
Urinary calculi within 30 d	138 (4.4)	35 (3.7)
Acute prostatitis within 30 d	15 (0.5)	7 (0.7)
Enterobacterales isolated from bloodstream		
* Escherichia coli*	2254 (71.9)	711 (74.5)
* Proteus mirabilis*	189 (6.0)	116 (12.1)
* Klebsiella pneumoniae*	589 (18.8)	104 (10.9)
* Klebsiella oxytoca*	75 (2.4)	14 (1.5)
Other or unspecified *Klebsiella* spp	14 (0.4)	5 (0.5)
Other or unspecified *Proteus* spp	13 (0.4)	5 (0.5)
Acute characteristics^b^		
Time from hospitalization to bacteremia ≥48 h	159 (5.1)	28 (2.9)
Antibiotic initiation		
Intensive care unit	543 (17.3)	165 (17.3)
Vasopressors	122 (3.9)	30 (3.1)
Serum leukocyte ≥12 000 cells/μL	2145 (68.4)	615 (64.4)
Temperature ≥38.3 °C	1799 (57.4)	542 (56.8)
Oral step-down therapy		
Intensive care unit	84 (2.7)	22 (2.3)
Serum leukocyte ≥12 000 cells/μL	480 (15.3)	130 (13.6)
Temperature ≥38.3 °C	81 (2.6)	15 (1.6)
Weight, median (IQR), kg	85 (73-100)	86 (74-102)
Creatinine clearance while receiving oral step-down therapy, median (IQR), mL/min^c^	62 (44-81)	58 (41-76)
Treatment characteristics		
Time to in vitro active antibiotics, median (IQR), h	12 (6-20)	13 (7-21)
1st day of oral antibiotics alone, median (IQR), d	4 (4-5)	5 (4-5)
Day 2	138 (4.4)	32 (3.4)
Day 3	474 (15.1)	121 (12.7)
Day 4	1027 (32.8)	303 (31.7)
Day 5	893 (28.5)	302 (31.6)
Day 6	602 (19.2)	197 (20.6)
Oral antibiotic with in vitro activity	3077 (98.2)	937 (98.1)
Unknown	34 (1.1)	12 (1.3)
Antibiotic duration, median (IQR), d		
Total	14 (12-16)	14 (12-16)
Oral	10 (9-13)	10 (8-12)

Abbreviation: IQR, interquartile range.

SI conversion factors: To convert leukocytes to ×10^9^/L, multiply by 0.001; and creatinine to micromoles per liter, multiply by 88.4.

^a^ Measured in the 365 days before blood culture collection date unless otherwise specified. Other timeframes are before blood culture collection date unless otherwise specified.

^b^ Antibiotic initiation refers to maximum values within 1 calendar day of the first day of active antibiotics. Oral step-down refers to the maximum value on the first day of oral step-down therapy or the maximum values for the last day prior to oral step-down therapy with a recorded measurement.

^c^ Creatinine clearance calculated on the day of oral switch as (140 − [age, y])/(serum creatinine, mg/dL).

### Statistical Analysis

We used overlap weighting, a variant of inverse probability of treatment weighting, to make treatment groups as similar as possible.^20,21^ Variables included in the overlap weights model were selected a priori using the literature and clinical judgment to identify risk factors associated with either recurrent bacteremia or mortality.^6,7,22,23,24^ The included variables were as follows: year of bacteremia, age, sex, individual comorbidities,^18^ immunosuppression, liver cirrhosis, history of UTI, prostate hypertrophy, prostate cancer, urolithiasis, acute prostatitis, urologic procedures, other structural urologic comorbidities (ie, retention, stricture, obstruction, or other major structural abnormalities), spinal cord injury or multiple sclerosis, nosocomial bacteremia (ie, onset ≥48 hours after hospital admission), intensive care unit status at blood culture collection and oral step-down, vasopressor receipt at antibiotic initiation, and presence of an elevated serum leukocyte count or fever at antibiotic initiation and oral step-down. Fever was defined as a temperature of 38.3 °C or greater.^3^ Elevated serum leukocyte count was defined as 12 000 cells/μL or greater. The median value was used for patients with missing values for leukocyte count or temperature. Diagnoses, procedures, and medications were assumed to be nonmissing if they were not coded in the medical record. Balance between groups before and after weighting was assessed using standardized mean differences and was considered similar if less than 0.10. Overlap weighting was performed using the PSW package and Overlap option in R, version 3.6.1 (R Foundation for Statistical Computing).^25^ Adjusted relative risks (aRRs) and adjusted risk differences (aRDs) for the association of treatment group with both primary and secondary outcomes were estimated using log-binomial regression with robust SEs to account for clustering within facilities. Both aRRs and aRDs were generated from augmented estimation in the PSW package in R, version 3.6.1, using the binomial outcome family with the same covariates specified in the overlap weighting model. A priori hypothesis tests were 2-sided, and results were considered statistically significant if 95% CIs did not cross 1.00 for aRRs and 0.00 for aRDs. Data collection and analyses were preplanned, except for the time-to-event analysis, which was performed as a descriptive analysis to explore for potential differences in time to outcomes (eAppendix in the Supplement).

## Results

### Study Population

A total of 4089 patients (3731 men [91.2%]; median age, 71 years [interquartile range, 63-81 years]) were included (Figure and Table 1); 955 received a β-lactam antibiotic, and 3134 received fluoroquinolones or TMP-SMX (2839 received fluoroquinolones and 295 received TMP-SMX). Exposure to β-lactam antibiotics and fluoroquinolones or TMP-SMX was approximately stable during the study period (eFigure 1 and eFigure 2 in the Supplement). Fewer than 20% of all patients were critically ill at the start of antibiotics based on presence in an intensive care unit (708 [17.3%]) or receipt of vasopressors (152 [3.7%]) (Table 1). In addition, patients were clinically stable at the time of oral step-down therapy based on intensive care unit status (106 [2.6%]), presence of fever (96 [2.0%]), and elevated serum leukocyte count (610 [14.9%]). The first day of oral antibiotics alone occurred at a median of 5 days (interquartile range, 4-5 days) for those in the oral β-lactam antibiotic group and 4 days (interquartile range, 4-5 days) for those who received fluoroquinolones or TMP-SMX. Overall, overlap weighting resulted in a well-balanced cohort (eFigure 3 and eFigure 4 in the Supplement).

**Figure.**
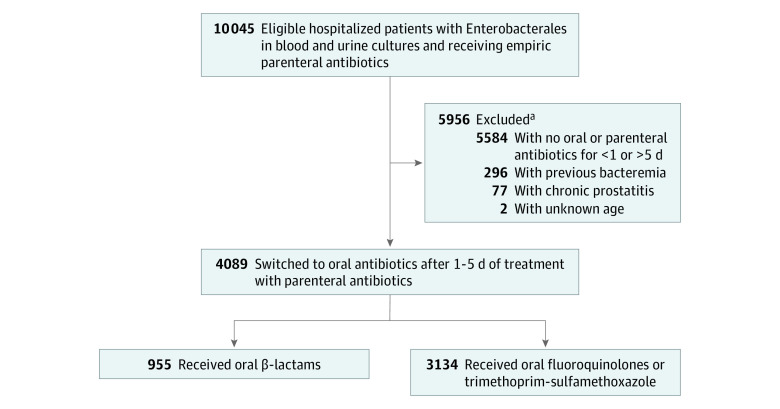
Flow Diagram of Inclusion, Exclusion, and Exposure Grouping ^a^Three patients met multiple exclusion criteria.

### Outcomes

The primary outcome, a composite of 30-day mortality or recurrent bacteremia, occurred for 42 patients (4.4%) in the oral β-lactam antibiotic group and for 94 patients (3.0%) who received fluoroquinolones or TMP-SMX (aRD, 0.99% [95% CI, −0.42% to 2.40%]; aRR, 1.31 [95% CI, 0.87-1.95]) (Table 2). Recurrent bacteremia within 30 days of switching to oral therapy occurred in 14 patients (1.5%) in the oral β-lactam antibiotic group and 12 patients (0.4%) who received fluoroquinolones or TMP-SMX (aRD, 1.03% [95% CI, 0.24%-1.82%]; aRR, 3.43 [95% CI, 0.42-27.90]). There were no significant differences in 30-day mortality (β-lactam antibiotics, 29 [3.0%] vs fluoroquinolones or TMP-SMX, 82 [2.6%]; aRD, 0.06% [95% CI, −1.13% to 1.26%]; aRR, 1.02 [95% CI, 0.67-1.56]). Recurrent bacteremia within 90 days of switching to oral therapy occurred in 25 patients (2.6%) in the oral β-lactam antibiotic group and 34 patients (1.1%) who received fluoroquinolones or TMP-SMX (aRD, 1.38% [95% CI, 0.30%-2.47%]; aRR, 2.15 [95% CI, 0.92-5.01]). Outcomes by antibiotic are presented in Table 3. The time to primary outcome results are presented in the eAppendix, eFigure 5, and eFigure 6 in the Supplement.

**Table 2.** Outcomes

Outcome	Patients, No. (%)		aRD, % (95% CI)^a^	aRR (95% CI)^a^
	Fluoroquinolones or trimethoprim-sulfamethoxazole (n = 3134)	β-Lactam antibiotics (n = 955)		
30-d Mortality and recurrent bacteremia	94 (3.0)	42 (4.4)	0.99 (−0.42 to 2.40)	1.31 (0.87 to 1.95)
Mortality	82 (2.6)	29 (3.0)	0.06 (−1.13 to 1.26)	1.02 (0.67 to 1.56)
Recurrent bacteremia	12 (0.4)	14 (1.5)	1.03 (0.24 to 1.82)	3.43 (0.42 to 27.90)
90-d Mortality and recurrent bacteremia	238 (7.6)	96 (10.1)	1.81 (−0.24 to 3.87)	1.23 (0.96 to 1.56)
Mortality	208 (6.6)	75 (7.9)	0.68 (−1.16 to 2.52)	1.10 (0.85 to 1.42)
Recurrent bacteremia	34 (1.1)	25 (2.6)	1.38 (0.30 to 2.47)	2.15 (0.92 to 5.01)
Repeated hospitalization with UTI				
At 30 d	22 (0.7)	14 (1.5)	0.81 (−0.06 to 1.67)	2.08 (0.72 to 5.99)
At 90 d	46 (1.5)	29 (3.0)	1.46 (0.28 to 2.64)	1.94 (0.97 to 3.85)

Abbreviation: aRD, adjusted risk difference; aRR, adjusted relative risk; UTI, urinary tract infection.

^a^ Risk difference and relative risk calculated with fluoroquinolones or trimethoprim-sulfamethoxazole as the reference group and β-lactam antibiotics as the intervention group.

**Table 3.** Oral Step-Down Antibiotic Distribution, Dosing Regimens, and Outcomes

Drug	Patients, No. (%)	30-d Recurrent bacteremia, No./total No. (%)	30-d Mortality, No./total No. (%)	Dose, mg/dose^a^	Doses per day, No.	Patients, No./total No. (%)
**β-Lactam antibiotics (n = 955)**						
Amoxicillin-clavulanate potassium	251 (26.3)	4/251 (1.6)	13/251 (5.2)	875-125	2	161/251 (64.1)
				500-125	2	46/251 (18.3)
				500-125	3	28/251 (11.2)
Cephalexin	245 (25.7)	0	5/245 (2.0)	500	4	115/245 (46.9)
				500	2	57/245 (23.3)
				500	3	47/245 (19.2)
Cefpodoxime proxetil	243 (25.4)	4/243 (1.6)	8/243 (3.3)	200	2	154/243 (63.4)
				400	2	47/243 (19.3)
Cefuroxime sodium	97 (10.2)	2/97 (2.1)	0	500	2	83/97 (85.6)
				250	2	12/97 (12.4)
Amoxicillin	63 (6.6)	3/63 (4.8)	1/63 (1.6)	500	3	44/63 (69.8)
				500	2	9/63 (14.3)
Cefdinir	35 (3.7)	1/35 (2.9)	0	300	2	33/35 (94.3)
Cefixime	14 (1.5)	0	0	400	1	11/14 (78.6)
				400	2	3/14 (21.4)
Ampicillin sodium	6 (0.6)	0	2/6 (33.3)	500	4	2/6 (33.3)
				500	2	2/6 (33.3)
Cefadroxil	1 (0.1)	0	0	1000	1	1 (100)
**Fluoroquinolones or trimethoprim-sulfamethoxazole (n = 3134)**						
Ciprofloxacin	2447 (78.1)	9/2447 (0.4)	61/2447 (2.5)	500	2	2003/2447 (81.9)
				500	1	172/2447 (7.0)
				250	2	130/2447 (5.3)
				750	2	122/2447 (5.0)
Levofloxacin	374 (11.9)	0	13/374 (3.5)	750	1	156/374 (41.7)
				500	1	154/374 (41.2)
				250	1	43/374 (11.5)
Trimethoprim-sulfamethoxazole	295 (9.4)	3/295 (1.0)	7/295 (2.4)	800-160	2	259/295 (87.8)
Moxifloxacin hydrochloride	18 (0.6)	0	1/18 (5.6)	400	1	18 (100)

^a^ Specific dosing regimens accounting for 10% or more of each dose per drug are listed. All other unlisted doses were less than 10%. Each patient’s most common dosing regimen during index treatment course is reported.

In the 30 days after starting oral step-down therapy, repeated hospitalization with UTI occurred for 14 patients (1.5%) in the oral β-lactam antibiotic group and 22 patients (0.7%) who received fluoroquinolones or TMP-SMX (aRD, 0.81% [95% CI, −0.06% to 1.67%]; aRR, 2.08 [95% CI, 0.72-5.99]) (Table 2). During the initial antibiotic treatment course, 70 patients (7.3%) in the oral β-lactam antibiotic group and 228 patients (7.3%) who received fluoroquinolones or TMP-SMX were switched back to a parenteral antibiotic, for any reason, after day 1 of oral step-down therapy. The median time to switch back to parenteral antibiotics after day 1 of oral step-down therapy was 3 days (interquartile range, 1-8 days) for patients in the β-lactam antibiotic group and 3 days (interquartile range, 1-7 days) for those who received fluoroquinolones or TMP-SMX.

## Discussion

In this study of 4089 patients with *E coli*, *Klebsiella* spp, or *Proteus* spp bacteremia from a suspected urine source, step-down treatment with oral β-lactam antibiotics was not associated with a statistically significant higher relative risk of recurrent bacteremia compared with fluoroquinolones or TMP-SMX, and the absolute risk and risk difference were small (ie, aRD, 1.03% [95% CI, 0.24%-1.82%] at 30 days and 1.38% [95% CI, 0.30%-2.47%] at 90 days). There were no significant differences in mortality between the groups. These data suggest that oral β-lactam antibiotics are a reasonable step-down treatment option in the setting of Enterobacterales bacteremia from a urine source after initial parenteral antibiotics, primarily when alternative treatments are limited by antibiotic resistance or potential adverse effects.

Although there were 4089 patients, there were only 26 recurrent bacteremia events at 30 days and 59 at 90 days, limiting statistical power, as evidenced by the wide 95% CIs. All estimates suggest a higher risk of mortality or recurrent bacteremia with β-lactam antibiotics; these results would likely meet traditional thresholds for statistically significant differences given additional events. Our interpretation is then based on our assessment that a 1% to 3% risk difference in recurrent bacteremia, in the context of less than 3% overall risk, is not a clinically meaningful difference, even if these estimates met traditional thresholds for statistical significance. This assessment warrants further discussion. To our knowledge, there is no clear precedence to define a clinically meaningful difference from the infectious diseases literature, especially related to gram-negative bacteremia. The US Food and Drug Administration recommends a 10% noninferiority margin for clinical trials assessing drugs for complicated UTIs^26^; this effect size has been used in 2 recent trials comparing different antibiotic durations for gram-negative bacteremia.^27,28^ Some authors believe that adequate power to detect smaller effect sizes would be more appropriate for gram-negative bacteremia.^29^ Other published and ongoing trials used effect sizes of 5% and 4% to define noninferiority.^30,31^ Our estimates are below these thresholds, which we highlight for context, acknowledging differences in the study questions and designs. Individual clinicians may or may not accept this risk depending on the clinical context, but the estimates may help clinicians assess the risk of an undesirable outcome and inform decision-making.

To our knowledge, this is the largest cohort comparing oral step-down antibiotic selection for treatment of Enterobacterales bacteremia. We are not aware of any randomized clinical trials evaluating the role of oral β-lactam antibiotics as step-down therapy specifically for bacteremia. Oral β-lactam antibiotics alone without preceding parenteral antibiotics have been shown to be associated with higher clinical recurrence rates in UTI trials with or without bacteremia.^2,32,33^ In UTI trials in which β-lactam antibiotics were used as step-down therapy after initial intravenous antibiotics, clinical cure rates were either greater than 90% or did not differ between patients with and patients without bacteremia, but this observation was limited by the small number of patients with bacteremia.^34,35,36,37,38,39,40,41^ Several retrospective studies have more directly compared oral β-lactam antibiotics with established treatments for step-down therapy. Punjabi and colleagues^16^ performed a meta-analysis of 8 retrospective studies comparing β-lactam antibiotics with fluoroquinolones or TMP-SMX as step-down therapy in 2889 patients. Consistent with our results, there were no significant differences in mortality, but there was a nonstatistically significant higher odds of recurrent bacteremia with β-lactam antibiotics (1.9%) vs fluoroquinolones or TMP-SMX (1.0%; odds ratio, 2.12 [95% CI, 0.92-4.87]). Punjabi and colleagues^16^ also found that oral β-lactam antibiotics were associated with higher odds of any recurrent infection compared with fluoroquinolones or TMP-SMX (5.5% vs 2.0%; odds ratio, 2.06 [95% CI, 1.18-3.61]).^16^

There were notable differences between our study and prior studies. Nearly all of the previous studies included patients with several sources of bacteremia. We limited the likely source of bacteremia to urine and limited the organisms to select Enterobacterales against which oral β-lactam antibiotics typically have some in vitro activity. The urine source of bacteremia was associated with lower mortality and recurrent bacteremia rates in previous studies; therefore, our results may not apply to other sources of bacteremia and do not apply to other Enterobacterales species.^6,24^ Our population comprised nearly all older men, which represents a group at increased risk of recurrent infection because of functional and structural urologic abnormalities.^42^ However, the findings may not be applicable to younger patients in whom β-lactam antibiotics may result in subtherapeutic concentrations owing to higher renal clearance.^15^ Some authors recommend a 90-day outcome period, and the comparative considerations of different outcome windows have been discussed elsewhere.^43,44,45^

There are additional considerations for interpreting and applying our results. Existing breakpoints for oral β-lactam antibiotics are not intended to guide the treatment of gram-negative bloodstream infections.^46^ Even when reported as susceptible, oral β-lactam antibiotics given at traditional doses may result in subtherapeutic serum concentrations when organism minimum inhibitory concentrations are at the upper range of susceptibility.^8,15^ Therefore, individual patient characteristics associated with oral β-lactam pharmacokinetics, measured or predicted organism minimum inhibitory concentrations, and limitations of alternative treatments should be considered before the selection of an oral β-lactam antibiotic. Involvement of infectious diseases and antimicrobial stewardship programs should be considered to optimize patient selection.^47^ Another important consideration is that the shortest effective treatment duration is unknown, as durations shorter than 7 days have not been systematically studied, to our knowledge.^27,28^ Therefore, we may have overestimated the association of oral antibiotics with outcomes because it is plausible that some patients were adequately treated before initiating oral step-down therapy.^27,28^ Given these considerations, a better understanding of patient factors associated with recurrence is important, as patient-specific factors, rather than definitive antibiotic selection or route, may be the primary factors associated with suboptimal outcomes.

### Limitations

This study has some limitations. As previously discussed, our results are limited by inadequate statistical power so further study is warranted to validate these results. The VA health system is an integrated health system, so missing mortality data were likely minimal.^48^ Recurrent bacteremia or repeated hospitalizations with UTIs at non-VA hospitals would not have been captured. Rates of recurrent Enterobacterales bacteremia were similar to the 1.2% reported in recent observational studies but lower than the 3% in experimental studies, which also included nonurinary sources.^16,27,30^ Our definition of repeated hospitalization with UTI was different than recurrent infection without bacteremia in other studies.^16^ We were not able to verify the presence of urinary symptoms, so we included only hospitalized patients with microbiologic evidence and antibiotics targeting Enterobacterales to increase the likelihood of capturing the most clinically significant recurrences. This definition will miss recurrent UTIs without bacteremia outside the hospital and will capture treatment of asymptomatic bacteriuria.^49^ We do not expect the loss to follow-up or misclassification of the above end points to differ between exposure groups.

We assumed that matching results of urine and blood cultures indicated a urine source of bacteremia, which may have led to the inclusion of patients with nonurinary sources of bacteremia. Susceptibility testing was not always directly performed on the oral β-lactam antibiotics, so we used reported susceptibilities from agents with the same or narrower spectrum to make assumptions as to whether the oral β-lactam antibiotic had any potential in vitro activity. Last, we were not able to account for the presence or replacement of indwelling urinary catheters because there are no structured data on their use.^3^

## Conclusions

In this cohort study of adults with *E coli*, *Klebsiella* spp, or *Proteus* spp bacteremia from a suspected urine source, β-lactam antibiotics vs fluoroquinolones or TMP-SMX were not statistically significantly associated with a higher relative risk of recurrent bacteremia, and there was no difference in mortality. The absolute risk difference for recurrent bacteremia was small (ie, <3% at 30 and 90 days), suggesting that oral β-lactam antibiotics are a reasonable step-down treatment option on an individual patient basis, primarily when alternative options are limited by resistance or adverse effects. Additional research is needed given our statistical power limitations to identify the relative association of definitive antibiotic selection, as opposed to patient-specific factors, with outcomes.

## Notes

Supplement. **eAppendix.** Methods and Results **eTable.** Diagnosis and Procedure Codes for Urinary Tract Infection, Urologic Comorbidities, and Urologic Procedures **eFigure 1.** Total Bacteremia Cases by Year and Exposure **eFigure 2.** Annual Bacteremia Cases by Exposure **eFigure 3.** Distribution of Propensity Scores for Treatment Group **eFigure 4.** Standardized Mean Differences in Patient Characteristics Before and After Overlap Weighting **eFigure 5.** Kaplan-Meier Curve of Time to Mortality or Recurrent Bacteremia **eFigure 6.** Kaplan-Meier Curve of Time to Recurrent Bacteremia

## References

[1] Coburn B, Morris AM, Tomlinson G, Detsky AS Does this adult patient with suspected bacteremia require blood cultures? JAMA. 2012;308(5):502-511. doi:10.1001/jama.2012.8262 22851117

[2] Gupta K, Hooton TM, Naber KG, ; Infectious Diseases Society of America; European Society for Microbiology and Infectious Diseases International clinical practice guidelines for the treatment of acute uncomplicated cystitis and pyelonephritis in women: a 2010 update by the Infectious Diseases Society of America and the European Society for Microbiology and Infectious Diseases. Clin Infect Dis. 2011;52(5):e103-e120. doi:10.1093/cid/ciq257 21292654

[3] Hooton TM, Bradley SF, Cardenas DD, ; Infectious Diseases Society of America Diagnosis, prevention, and treatment of catheter-associated urinary tract infection in adults: 2009 international clinical practice guidelines from the Infectious Diseases Society of America. Clin Infect Dis. 2010;50(5):625-663. doi:10.1086/650482 20175247

[4] Mombelli G, Pezzoli R, Pinoja-Lutz G, Monotti R, Marone C, Franciolli M Oral vs intravenous ciprofloxacin in the initial empirical management of severe pyelonephritis or complicated urinary tract infections: a prospective randomized clinical trial. Arch Intern Med. 1999;159(1):53-58. doi:10.1001/archinte.159.1.53 9892331

[5] Eliakim-Raz N, Yahav D, Paul M, Leibovici L Duration of antibiotic treatment for acute pyelonephritis and septic urinary tract infection—7 days or less versus longer treatment: systematic review and meta-analysis of randomized controlled trials. J Antimicrob Chemother. 2013;68(10):2183-2191. doi:10.1093/jac/dkt177 23696620

[6] Tamma PD, Conley AT, Cosgrove SE, ; Antibacterial Resistance Leadership Group Association of 30-day mortality with oral step-down vs continued intravenous therapy in patients hospitalized with Enterobacteriaceae bacteremia. JAMA Intern Med. 2019;179(3):316-323. doi:10.1001/jamainternmed.2018.6226 30667477 PMC6439703

[7] Kutob LF, Justo JA, Bookstaver PB, Kohn J, Albrecht H, Al-Hasan MN Effectiveness of oral antibiotics for definitive therapy of gram-negative bloodstream infections. Int J Antimicrob Agents. 2016;48(5):498-503. doi:10.1016/j.ijantimicag.2016.07.013 27590704

[8] Mogle BT, Beccari MV, Steele JM, Fazili T, Kufel WD Clinical considerations for oral beta-lactams as step-down therapy for Enterobacteriaceae bloodstream infections. Expert Opin Pharmacother. 2019;20(8):903-907. doi:10.1080/14656566.2019.1594774 30908107

[9] Diallo K, Thilly N, Luc A, ; ESGAP, ESGBIS Management of bloodstream infections by infection specialists: an international ESCMID cross-sectional survey. Int J Antimicrob Agents. 2018;51(5):794-798. doi:10.1016/j.ijantimicag.2017.12.010 29309899

[10] Hospenthal DR, Waters CD, Beekmann SE, Polgreen PM Practice patterns of infectious diseases physicians in transitioning from intravenous to oral therapy in patients with bacteremia. Open Forum Infect Dis. 2019:ofz386. doi:10.1093/ofid/ofz386 PMC7731529 33335941

[11] Al-Hasan MN, Rac H Transition from intravenous to oral antimicrobial therapy in patients with uncomplicated and complicated bloodstream infections. Clin Microbiol Infect. 2020;26(3):299-306. doi:10.1016/j.cmi.2019.05.012 31128289

[12] US Food and Drug Administration. FDA drug safety communication: FDA updates warnings for oral and injectable fluoroquinolone antibiotics due to disabling side effects. Published May 12, 2016. Accessed March 15, 2020. https://www.fda.gov/Drugs/DrugSafety/ucm511530.htm

[13] Morrill HJ, Morton JB, Caffrey AR, Antimicrobial resistance of *Escherichia coli* urinary isolates in the Veterans Affairs health care system. Antimicrob Agents Chemother. 2017;61(5):e02236-16. doi:10.1128/AAC.02236-16 28193660 PMC5404570

[14] Keller SC, Williams D, Gavgani M, Rates of and risk factors for adverse drug events in outpatient parenteral antimicrobial therapy. Clin Infect Dis. 2018;66(1):11-19. doi:10.1093/cid/cix733 29020202 PMC5848264

[15] Cattrall JWS, Asín-Prieto E, Freeman J, Trocóniz IF, Kirby A A pharmacokinetic–pharmacodynamic assessment of oral antibiotics for pyelonephritis. Eur J Clin Microbiol Infect Dis. 2019;38(12):2311-2321. doi:10.1007/s10096-019-03679-9 31494827 PMC6858297

[16] Punjabi C, Tien V, Meng L, Deresinski S, Holubar M Oral fluoroquinolone or trimethoprim-sulfamethoxazole vs. β-lactams as step-down therapy for Enterobacteriaceae bacteremia: systematic review and meta-analysis. Open Forum Infect Dis. 2019;6(10):ofz364. doi:10.1093/ofid/ofz364 31412127 PMC6785705

[17] Daneman N, Rishu AH, Xiong W, ; Canadian Critical Care Trials Group Bacteremia Antibiotic Length Actually Needed for Clinical Effectiveness (BALANCE): study protocol for a pilot randomized controlled trial. Trials. 2015;16(1):173. doi:10.1186/s13063-015-0688-z 25903783 PMC4407544

[18] Gagne JJ, Glynn RJ, Avorn J, Levin R, Schneeweiss S A combined comorbidity score predicted mortality in elderly patients better than existing scores. J Clin Epidemiol. 2011;64(7):749-759. doi:10.1016/j.jclinepi.2010.10.004 21208778 PMC3100405

[19] Drekonja DM, Rector TS, Cutting A, Johnson JR Urinary tract infection in male veterans: treatment patterns and outcomes. JAMA Intern Med. 2013;173(1):62-68. doi:10.1001/2013.jamainternmed.829 23212273

[20] Li F, Thomas LE, Li F Addressing extreme propensity scores via the overlap weights. Am J Epidemiol. 2019;188(1):250-257. doi:10.1093/aje/kwy201 30189042

[21] Thomas LE, Li F, Pencina MJ Overlap weighting: a propensity score method that mimics attributes of a randomized clinical trial. JAMA. 2020;323(23):2417-2418. doi:10.1001/jama.2020.7819 32369102

[22] Mercuro NJ, Stogsdill P, Wungwattana M Retrospective analysis comparing oral stepdown therapy for Enterobacteriaceae bloodstream infections: fluoroquinolones versus β-lactams. Int J Antimicrob Agents. 2018;51(5):687-692. doi:10.1016/j.ijantimicag.2017.12.007 29284155

[23] Giannella M, Pascale R, Toschi A, Treatment duration for *Escherichia coli* bloodstream infection and outcomes: retrospective single-centre study. Clin Microbiol Infect. 2018;24(10):1077-1083. doi:10.1016/j.cmi.2018.01.013 29371138

[24] Sousa A, Pérez-Rodríguez MT, Suárez M, Short- versus long-course therapy in gram-negative bacilli bloodstream infections. Eur J Clin Microbiol Infect Dis. 2019;38(5):851-857. doi:10.1007/s10096-019-03467-5 30680566

[25] Mao H, Li L. PSW: propensity score weighting methods for dichotomous treatments. R package version 1.1-3. Published 2018. Accessed March 15, 2020. https://cran.r-project.org/package=PSW

[26] US Food and Drug Administration Center for Drug Evaluation and Research Guidance document: complicated urinary tract infections: developing drugs for treatment. Published June 2018. Accessed June 23, 2020. https://www.fda.gov/regulatory-information/search-fda-guidance-documents/complicated-urinary-tract-infections-developing-drugs-treatment

[27] Yahav D, Franceschini E, Koppel F, ; Bacteremia Duration Study Group Seven versus 14 days of antibiotic therapy for uncomplicated gram-negative bacteremia: a noninferiority randomized controlled trial. Clin Infect Dis. 2019;69(7):1091-1098. doi:10.1093/cid/ciy1054 30535100

[28] von Dach E, Albrich WC, Brunel A-S, Effect of C-reactive protein–guided antibiotic treatment duration, 7-day treatment, or 14-day treatment on 30-day clinical failure rate in patients with uncomplicated gram-negative bacteremia: a randomized clinical trial. JAMA. 2020;323(21):2160-2169. doi:10.1001/jama.2020.6348 32484534 PMC7267846

[29] Daneman N, Fowler RA Shortening antibiotic treatment durations for bacteremia. Clin Infect Dis. 2019;69(7):1099-1100. doi:10.1093/cid/ciy1057 30535118

[30] Harris PNA, Tambyah PA, Lye DC, ; MERINO Trial Investigators and the Australasian Society for Infectious Disease Clinical Research Network (ASID-CRN) Effect of piperacillin-tazobactam vs meropenem on 30-day mortality for patients with *E coli* or *Klebsiella pneumoniae* bloodstream infection and ceftriaxone resistance: a randomized clinical trial. JAMA. 2018;320(10):984-994. doi:10.1001/jama.2018.12163 30208454 PMC6143100

[31] Daneman N, Rishu AH, Pinto R, ; Canadian Critical Care Trials Group 7 Versus 14 days of antibiotic treatment for critically ill patients with bloodstream infection: a pilot randomized clinical trial. Trials. 2018;19(1):111. doi:10.1186/s13063-018-2474-1 29452598 PMC5816399

[32] Stamm WE, McKevitt M, Counts GW Acute renal infection in women: treatment with trimethoprim-sulfamethoxazole or ampicillin for two or six weeks. a randomized trial. Ann Intern Med. 1987;106(3):341-345. doi:10.7326/0003-4819-106-3-341 3492950

[33] Sandberg T, Englund G, Lincoln K, Nilsson LG Randomised double-blind study of norfloxacin and cefadroxil in the treatment of acute pyelonephritis. Eur J Clin Microbiol Infect Dis. 1990;9(5):317-323. doi:10.1007/BF01973737 2197091

[34] Jernelius H, Zbornik J, Bauer CA One or three weeks’ treatment of acute pyelonephritis? a double-blind comparison, using a fixed combination of pivampicillin plus pivmecillinam. Acta Med Scand. 1988;223(5):469-477. doi:10.1111/j.0954-6820.1988.tb15899.x 3287839

[35] Johnson JR, Lyons MF II, Pearce W, Therapy for women hospitalized with acute pyelonephritis: a randomized trial of ampicillin versus trimethoprim-sulfamethoxazole for 14 days. J Infect Dis. 1991;163(2):325-330. doi:10.1093/infdis/163.2.325 1988516

[36] Cronberg S, Banke S, Bruno AM, Ampicillin plus mecillinam vs. cefotaxime/cefadroxil treatment of patients with severe pneumonia or pyelonephritis: a double-blind multicentre study evaluated by intention-to-treat analysis. Scand J Infect Dis. 1995;27(5):463-468. doi:10.3109/00365549509047047 8588136

[37] Millar LK, Wing DA, Paul RH, Grimes DA Outpatient treatment of pyelonephritis in pregnancy: a randomized controlled trial. Obstet Gynecol. 1995;86(4, pt 1):560-564. doi:10.1016/S0029-7844(95)80016-6 7675380

[38] Sandberg T, Alestig K, Eilard T, Aminoglycosides do not improve the efficacy of cephalosporins for treatment of acute pyelonephritis in women. Scand J Infect Dis. 1997;29(2):175-179. doi:10.3109/00365549709035880 9181655

[39] Moreno-Martínez A, Mensa J, Martínez JA, Cefixime versus amoxicillin plus netilmicin in the treatment of community-acquired non-complicated acute pyelonephritis. Article in Spanish. Med Clin (Barc). 1998;111(14):521-524. 9859076

[40] Sanchez M, Collvinent B, Miró O, Short-term effectiveness of ceftriaxone single dose in the initial treatment of acute uncomplicated pyelonephritis in women: a randomised controlled trial. Emerg Med J. 2002;19(1):19-22. doi:10.1136/emj.19.1.19 11777865 PMC1725780

[41] Monmaturapoj T, Montakantikul P, Mootsikapun P, Tragulpiankit P A prospective, randomized, double dummy, placebo-controlled trial of oral cefditoren pivoxil 400 mg once daily as switch therapy after intravenous ceftriaxone in the treatment of acute pyelonephritis. Int J Infect Dis. 2012;16(12):e843-e849. doi:10.1016/j.ijid.2012.07.009 22951426

[42] Schaeffer AJ, Nicolle LE Urinary tract infections in older men. N Engl J Med. 2016;374(22):2192. doi:10.1056/NEJMc1603508 27248641

[43] Harris PNA, McNamara JF, Lye DC, Proposed primary endpoints for use in clinical trials that compare treatment options for bloodstream infection in adults: a consensus definition. Clin Microbiol Infect. 2017;23(8):533-541. doi:10.1016/j.cmi.2016.10.023 27810466

[44] Al-Hasan MN, Albrecht H, Bookstaver PB, Kohn J, Justo JA Duration of antimicrobial therapy for Enterobacteriaceae bacteremia: using convenient end points for convenient conclusions. Clin Infect Dis. 2018;66(12):1978-1979. doi:10.1093/cid/ciy043 29365087

[45] Chotiprasitakul D, Han JH, Cosgrove SE, Harris AD, Lautenbach E, Tamma PD Reply to Al-Hasan et al. Clin Infect Dis. 2018;66(12):1979-1981. doi:10.1093/cid/ciy046 29365075

[46] Clinical and Laboratory Standards Institute M100: performance standards for antimicrobial susceptibility testing, 30th edition. Accessed September 2, 2020. https://clsi.org/standards/products/microbiology/documents/m100/

[47] Erickson RM, Tritle BJ, Spivak ES, Timbrook TT Impact of an antimicrobial stewardship bundle for uncomplicated gram-negative bacteremia. Open Forum Infect Dis. 2019;6(12):ofz490. doi:10.1093/ofid/ofz490 32128333 PMC7047945

[48] Sohn MW, Arnold N, Maynard C, Hynes DM Accuracy and completeness of mortality data in the Department of Veterans Affairs. Popul Health Metr. 2006;4:2. doi:10.1186/1478-7954-4-2 16606453 PMC1458356

[49] Spivak ES, Burk M, Zhang R, ; Management of Urinary Tract Infections Medication Use Evaluation Group Management of bacteriuria in Veterans Affairs hospitals. Clin Infect Dis. 2017;65(6):910-917. doi:10.1093/cid/cix474 28531289

