# Supplementary material for: Oral β-Lactam Antibiotics vs Fluoroquinolones or Trimethoprim-Sulfamethoxazole for Definitive Treatment of Enterobacterales Bacteremia From a Urine Source
Source: JAMA Netw Open. 2020 Oct 8;3(10):e2020166. doi: 10.1001/jamanetworkopen.2020.20166 (PMC7545306; doi:10.1001/jamanetworkopen.2020.20166)
Supplement: Supplement. — eAppendix. Methods and Results eTable. Diagnosis and Procedure Codes for Urinary Tract Infection, Urologic Comorbidities, and Urologic Procedures eFigure 1. Total Bacteremia Cases by Year and Exposure eFigure 2. Annual Bacteremia Cases by Exposure eFigure 3. Distribution of Propensity Scores for Treatment Group eFigure 4. Standardized Mean Differences in Patient Characteristics Before and After Overlap Weighting eFigure 5. Kaplan-Meier Curve of Time to Mortality or Recurrent Bacteremia eFigure 6. Kaplan-Meier Curve of Time to Recurrent Bacteremia [file jamanetwopen-e2020166-s001.pdf]

Supplementary Online Content

Sutton JD, Stevens VW, Chang NCN, Khader K, Timbrook TT, Spivak ES. Oral β-lactam antibiotics vs fluoroquinolones or trimethoprim-sulfamethoxazole for definitive treatment of Enterobacterales bacteremia from a urine source. *JAMA Netw Open.* 2020;3(10):e2020166. doi:10.1001/jamanetworkopen.2020.20166

eAppendix. Methods and Results

eTable. Diagnosis and Procedure Codes for Urinary Tract Infection, Urologic Comorbidities, and Urologic Procedures

eFigure 1. Total Bacteremia Cases by Year and Exposure

eFigure 2. Annual Bacteremia Cases by Exposure

eFigure 3. Distribution of Propensity Scores for Treatment Group

eFigure 4. Standardized Mean Differences in Patient Characteristics Before and After Overlap Weighting

eFigure 5. Kaplan-Meier Curve of Time to Mortality or Recurrent Bacteremia

eFigure 6. Kaplan-Meier Curve of Time to Recurrent Bacteremia

This supplementary material has been provided by the authors to give readers additional information about their work.

© 2020 Sutton JD et al. *JAMA Network Open.*

eAppendix. Methods and Results

Methods

For repeat hospitalization with UTI within 30 or 90 days, Gram-negative antibiotics included any parenteral or oral aminoglycoside, beta-lactam, beta-lactam/beta-lactamase inhibitor, fluoroquinolone, polymyxin, trimethoprim with or without sulfamethoxazole, fosfomycin, or nitrofurantoin. Urine cultures had to be obtained in the four calendar days before or anytime during repeat hospitalization.

Further time-to-event analysis was performed post-hoc to describe and explore potential differences in time to outcomes. The unadjusted time to primary outcome was estimated using the Kaplan-Meier approach and log rank test. Cox Proportional Hazards models were used to estimate adjusted hazard ratios.

Results

The unadjusted median time to the primary outcome, mortality or recurrent bacteremia, was 33 days (IQR 18, 58) in the beta-lactam group and 37 days (IQR 23, 66) in FQ/TMP-SMX (eFigure 5). The adjusted Hazard Ratio for time to mortality or recurrent bacteremia was 1.28 (95% CI 0.99 – 1.63). The unadjusted median time to recurrent bacteremia was 28 days (IQR 21, 49) in the beta-lactam group and 37 days (IQR 28, 44) in FQ/TMP-SMX (eFigure 6). The adjusted hazard ratio for time to recurrent bacteremia was 2.25 (95% CI 1.32 – 3.86). The median time to mortality was 35 days (IQR 18, 58) in the beta-lactam group and 38 days (IQR 23, 68) in FQ/TMP-SMX.

© 2020 Sutton JD et al. *JAMA Network Open.*

eTable. Diagnosis and Procedure Codes for Urinary Tract Infection, Urologic Comorbidities, and Urologic Procedures

Diagnosis / Procedure	Code(s)
Urinary tract infection (outcome)	590.*, 595.*, 599.0, 966.4, 996.5
History of urinary tract infection (exposure)	590.00, 590.01, 590.10, 590.11, 590.80, 590.81, 590.9, 595.*, 599.0, 996.64, 996.65
Prostate hypertrophy	600.*
Urinary retention, obstruction, or other structural urologic abnormality	344.61, 591.*, 593.3, 593.4, 593.5, 593.70, 593.71, 593.72, 596.0, 596.1, 596.2, 596.3, 596.4, 596.53, 596.54, 596.55, 596.59, 596.6, 598.*, 599.1, 599.2, 599.4, 599.60, 599.61, 788.2*,788.3*,788.91, 996.31
Prostate cancer	185.*, V10.46
Spinal cord injury, paraplegia, quadriplegia, or multiple sclerosis	340.*, 344.0*, 344.1, 952.*
Urinary calculi	592.0, 592.1, 592.2, 594.0, 594.1, 594.2, 594.8, 594.9
Acute prostatitis	601.0
Urologic procedure (ICD-9-CM)[b, c]	55.*, 56.*, 57*, 58.*, 59*, 60*, 64*<br>Excluded codes: 57.32, 57.94, 57.95, 59.8
Urologic procedure (CPT)[c, d]	All codes in the categories ‘Urinary System’ and ‘Male Genital System’ except the following: 50300, 50320, 50323, 50325, 50327, 50328, 50329, 50547, 51000, 51005, 51010, 51100, 51101, 51102, 51700, 51701, 51702, 51703, 51705, 51710, 51725, 51736, 51795, 51797, 51798, 53670, 53675

[a] International Classification of Diseases, Ninth Revision diagnosis code (ICD-9-CM)
[b] ICD-9-CM Procedure Code
[c] Procedure codes for bladder catheter insertions, post-void residual measurements, and cystoscopies without biopsy or other intervention were excluded. The observed codes after the data pull were screened for these types of procedures.
[d] Current Procedural Terminology (CPT) codes

© 2020 Sutton JD et al. *JAMA Network Open.*

eFigure 1. Total Bacteremia Cases by Year and Exposure

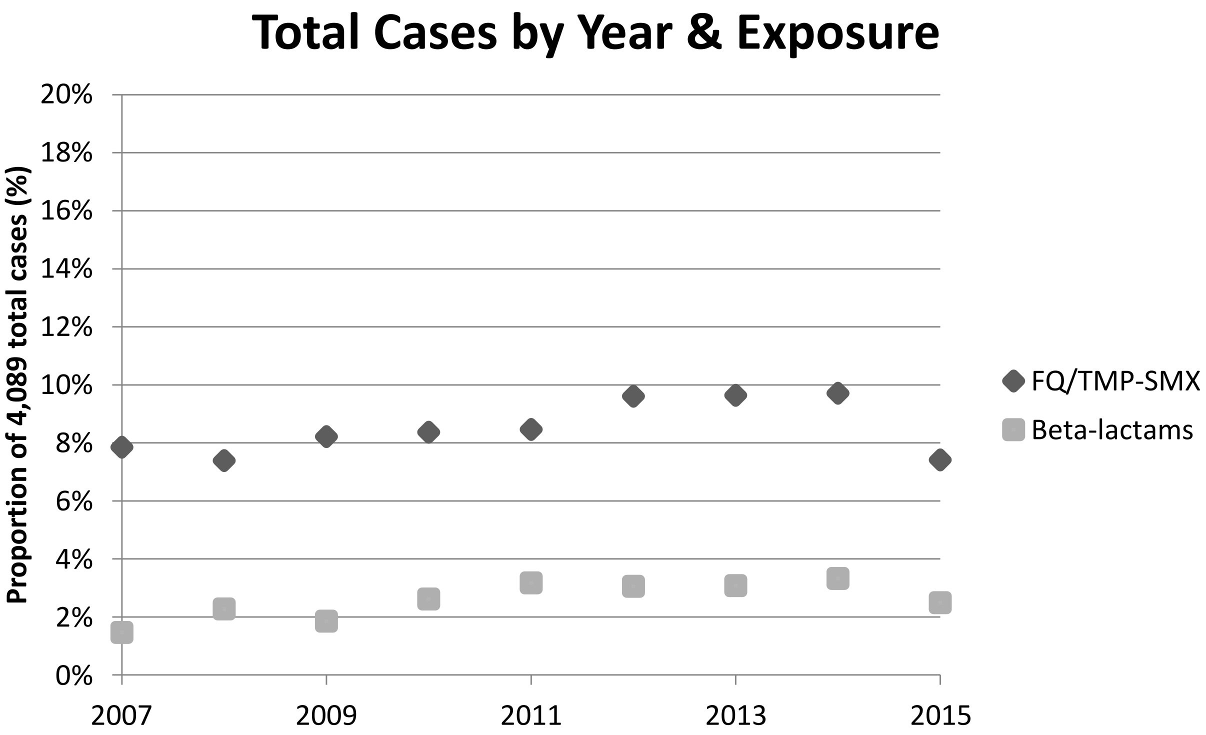

© 2020 Sutton JD et al. *JAMA Network Open.*

eFigure 2. Annual Bacteremia Cases by Exposure

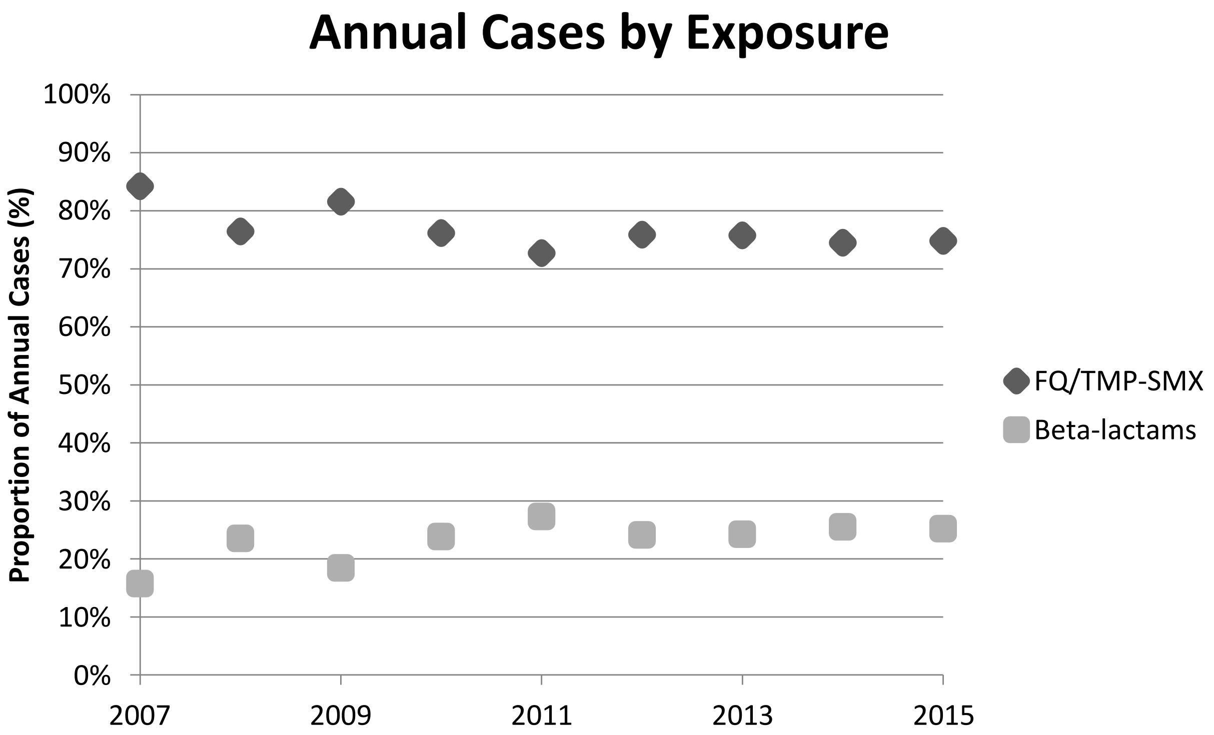

© 2020 Sutton JD et al. *JAMA Network Open.*

eFigure 3. Distribution of Propensity Scores for Treatment Group

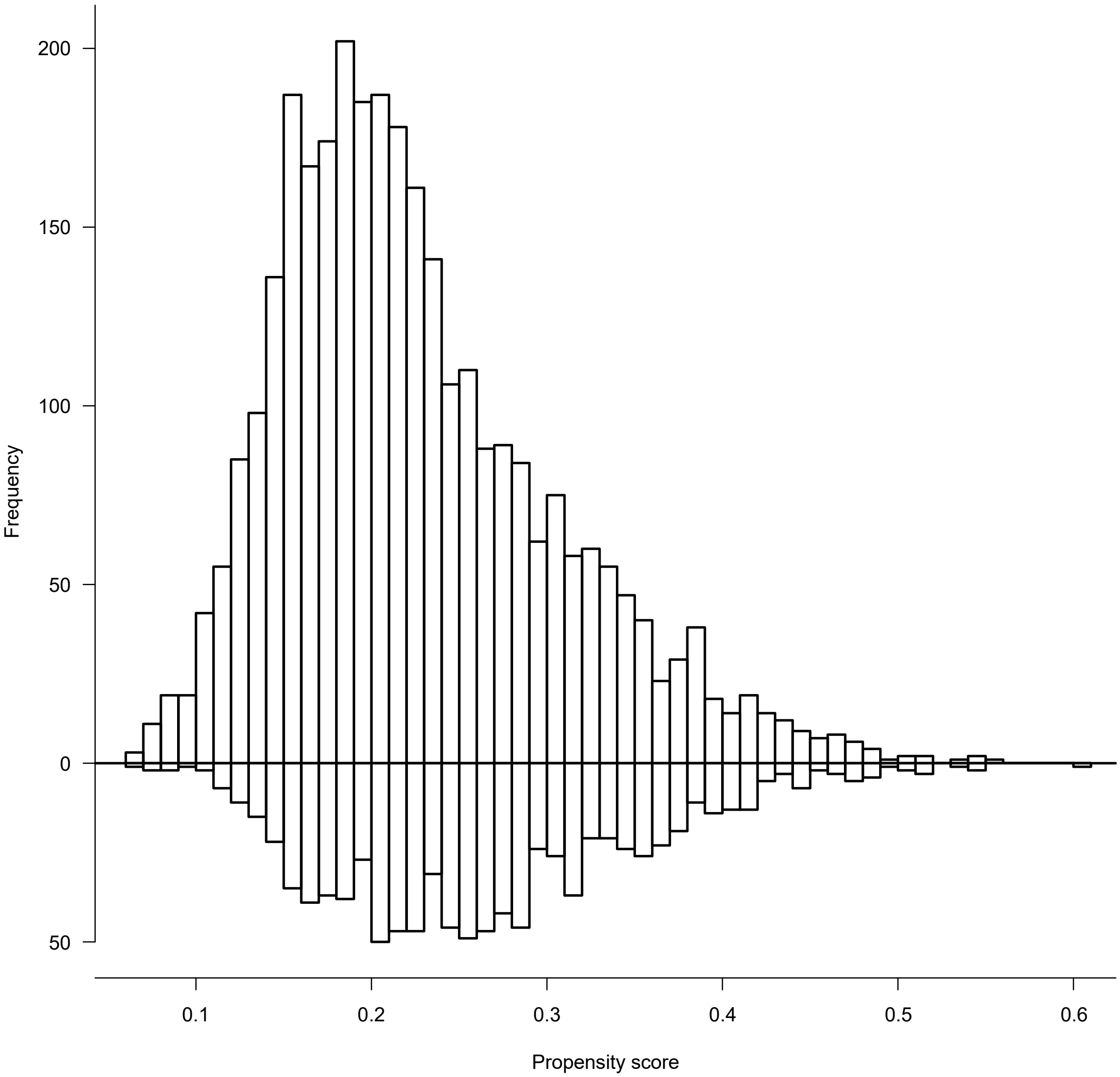

© 2020 Sutton JD et al. *JAMA Network Open.*

eFigure 4. Standardized Mean Differences in Patient Characteristics Before and After Overlap Weighting

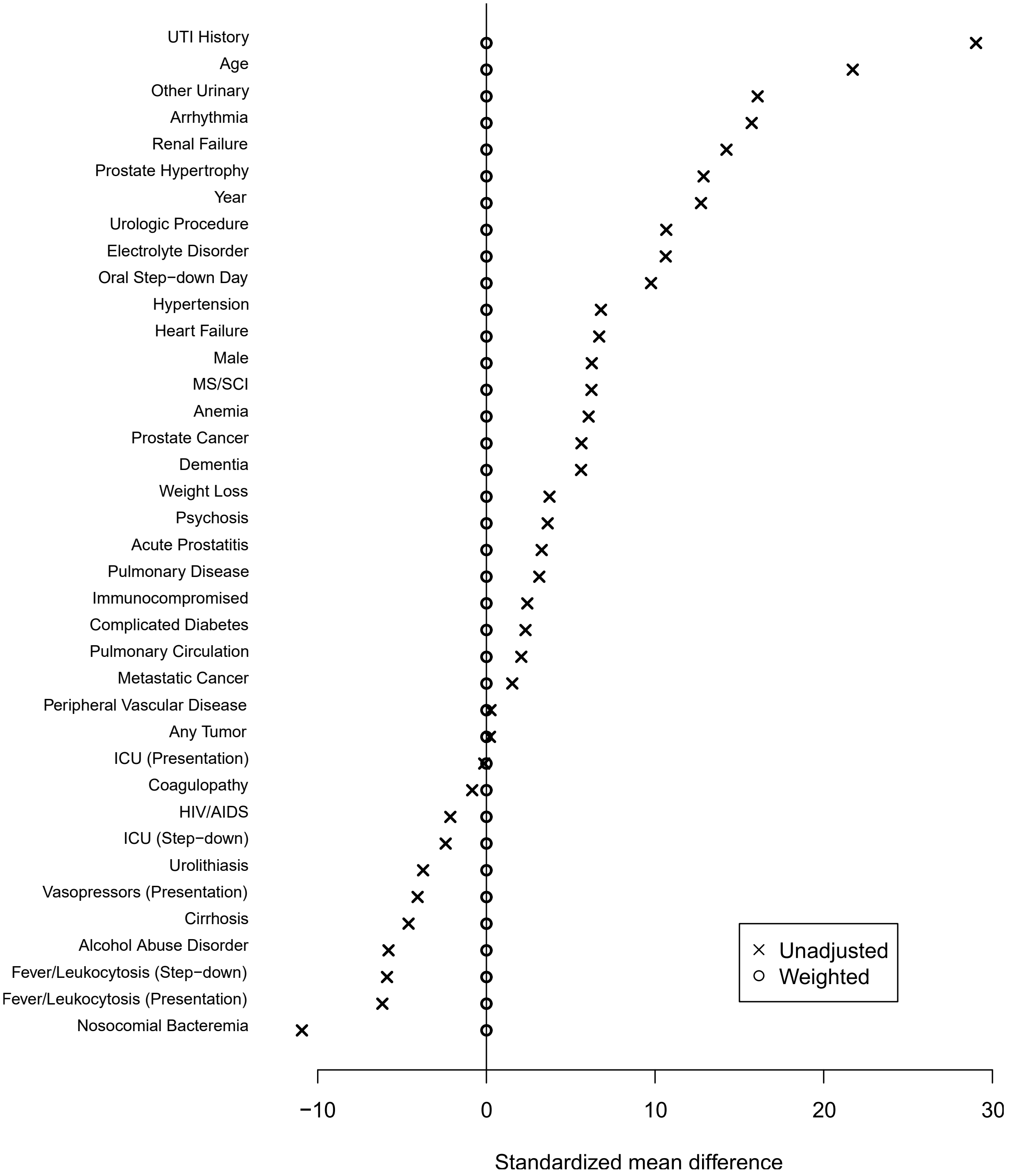

eFigure 4 Caption

UTI: urinary tract infection, MS/SCI: multiple sclerosis or spinal cord injury, ICU: intensive care unit, presentation: within one calendar day of the day of active antibiotics, step-down: day of oral step-down or the maximum values for the last day prior to oral step-down with a recorded measurement, fever: temperature ≥ 101˚F, leukocytosis: serum leukocyte count ≥ 12,000 / mm^3

© 2020 Sutton JD et al. *JAMA Network Open.*

eFigure 5. Kaplan-Meier Curve of Time to Mortality or Recurrent Bacteremia

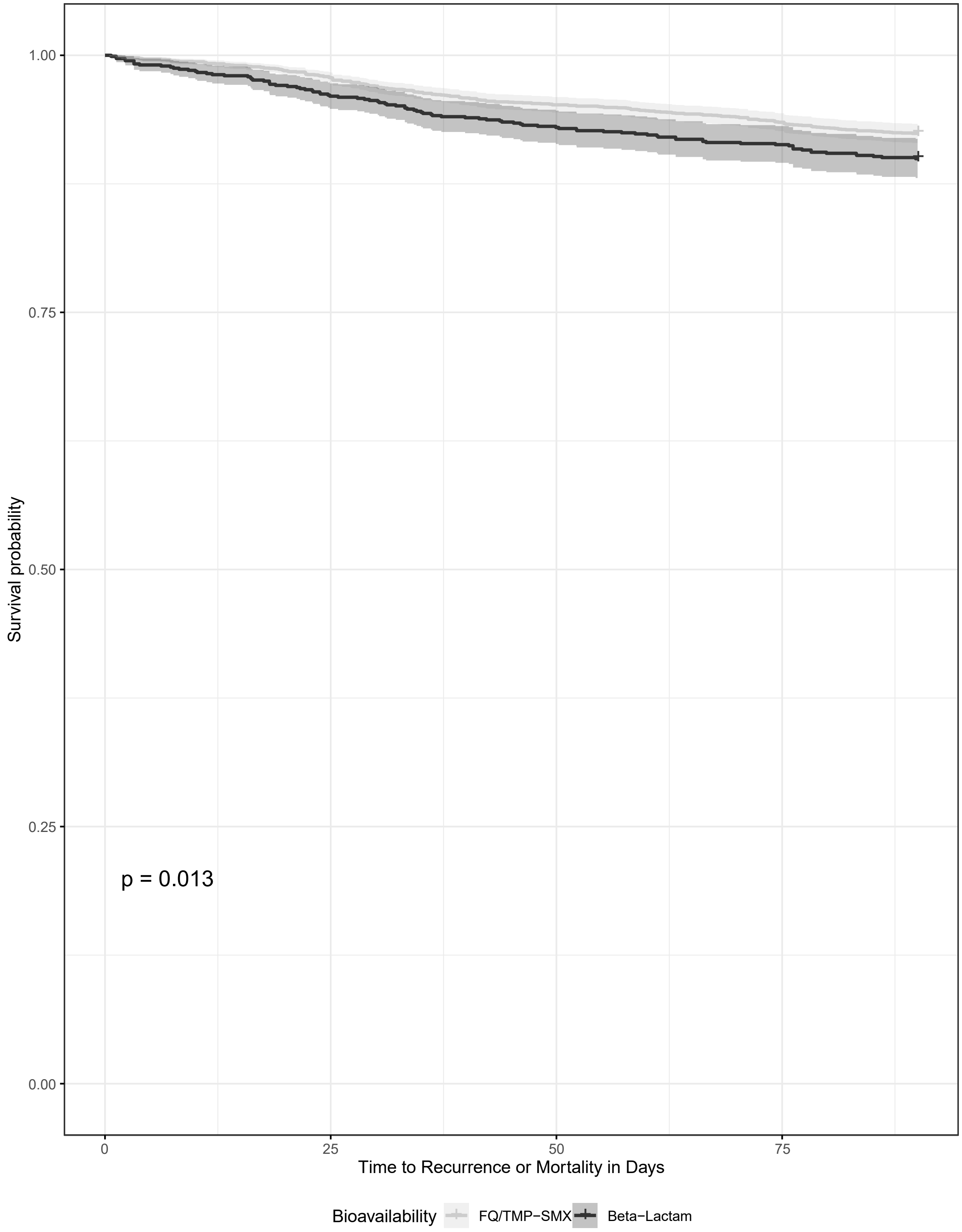

© 2020 Sutton JD et al. *JAMA Network Open.*

eFigure 6. Kaplan-Meier Curve of Time to Recurrent Bacteremia

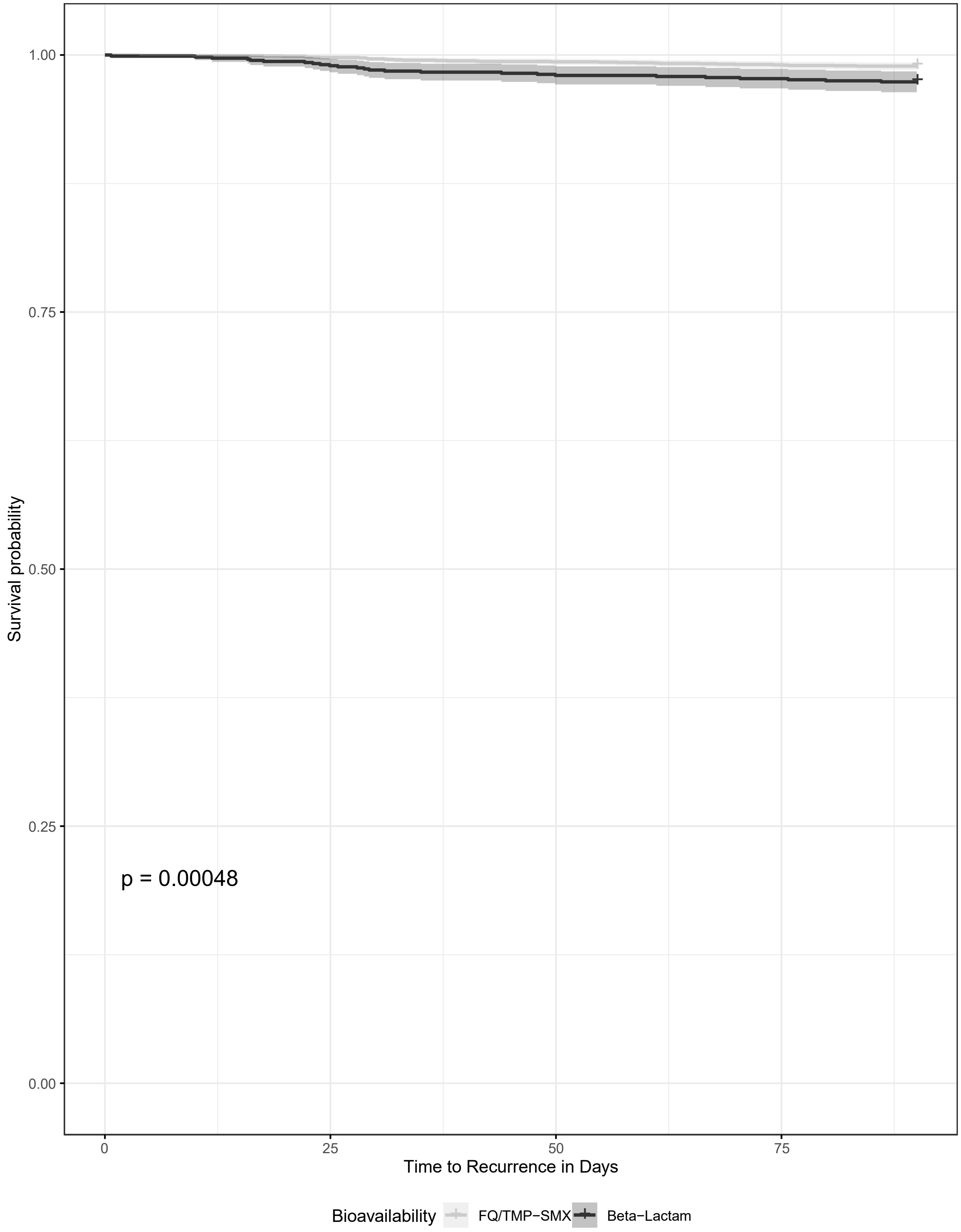

© 2020 Sutton JD et al. *JAMA Network Open.*
